# A high throughput multiplex PCR assay for simultaneous detection of seven aminoglycoside-resistance genes in Enterobacteriaceae

**DOI:** 10.1186/1471-2180-13-58

**Published:** 2013-03-14

**Authors:** Xiumei Hu, Banglao Xu, Yinmei Yang, Dayu Liu, Mengjie Yang, Ji Wang, Hongwei Shen, Xiaomian Zhou, Xuejun Ma

**Affiliations:** 1 Key Laboratory for Medical Virology, Ministry of Health, National Institute for Viral Disease Control and Prevention, Chinese Center for Disease Control and Prevention, Beijing 102206, People’s Republic of China; 2 Department of Laboratory Medicine, Nanfang Hospital, Southern Medical University, Guangzhou 510515, People’s Republic of China; 3 Department of Laboratory Medicine, Guangzhou First Municipal People’s Hospital, Affiliated Hospital of Guangzhou Medical College, Guangzhou 510180, People’s Republic of China

**Keywords:** Aminoglycosides, Resistance genes, GeXP analyzer, Multiplex PCR, Capillary electrophoresis

## Abstract

**Background:**

The aminoglycoside-resistance genes encoding aminoglycoside modifying enzymes and 16S rRNA methyltransferases are main factors contributing to increasing resistance to aminoglycosides. Characterization and distribution of antimicrobial resistance gene profiles provide important information on the potential difficulty of treatment of bacteria. Several molecular methods have been developed to investigate the prevalence of aminoglycoside-resistance genes. These existing methods are time-consuming, labor-intensive, expensive or limited sensitivity in the epidemiological investigation. Therefore, it is necessary to develop a rapid, less-costly and high throughput and sensitive method to investigate the distribution of antimicrobial resistance gene in clinical isolates.

**Results:**

In this study, we developed a GeXP analyzer-based multiplex PCR assay to simultaneously detect seven aminoglycoside-resistance genes, including *aac(3)-II*, *aac(6′)*-Ib, *aac(6′)-II*, *ant(3″)-I,aph(3′)-VI,armA* and *rmtB*, and to analyze the distribution of these genes in clinical Enterobacteriaceae isolates. Under optimized conditions, this assay achieved a limit-of-detection as low as 10 copies of each of the seven genes. The presented method was applied to analyze the distribution of aminoglycoside-resistance genes in 56 clinical Enterobacteriaceae isolates, and the results were compared with that of the conventional single PCR assay. Kappa values of the two methods for detecting each of the seven resistance genes were 0.831, 0.846, 0.810, 0.909, 0.887, 0.810 and 0.825, respectively.

**Conclusion:**

This GeXP assay is demonstrated to be a rapid, cost-effective and high throughput method with high sensitivity and specificity for simultaneously detecting seven common aminoglycoside-resistance genes.

## Background

Aminoglycosides are potent bactericidal antibiotics targeting the bacterial ribosome, where they bind to the A-site and disrupt protein synthesis. They are particularly active against aerobic, Gram-negative bacteria and act synergistically against certain Gram-positive organisms [1-3]. Unfortunately, their efficacy has been reduced by the surge and dissemination of resistance. In some cases the levels of resistance reached the point that rendered them virtually useless [4]. There are several considerable mechanisms that cause resistance to aminoglycosides including: 1) the acquisition of modifying enzymes such as acetyltransferases, phosphotransferases and adenylyltransferases, 2) modification of the target by mutation in ribosomal proteins [5] or in 16S rRNA [6], or by 16S rRNA methyltransferase such as ArmA [7], Rmt families [8,9] and NpmA [10], 3) decreased intracellular accumulation of the antibiotic by alteration of outer membrane permeability, diminished inner membrane transport, or active efflux pump [11]. The main factors contributing to the increasing isolates resistant to aminoglycosides were reported to be the widespread genes encoding aminoglycoside-modifying enzymes and 16S rRNA methyltransferase [2,12-14]. The most common aminoglycoside-modifying enzyme gene types are *aac(3)-II*, followed by *aac(6′)-I*, *ant(3″)-I*, *aph(3′)-II*, and *ant(2″)-I* in *Escherichia coli*[15]. Furthermore, *aac(6′)-II* and *aph(3′)-VI* are respectively the significant resistance determinants of gentamicin, tobramycin, and amikacin in *Pseudomonas aeruginosa*[4,16]. In addition, modification of 16S rRNA by methylases reduces binding to aminoglycosides, leading to high-level resistance to amikacin, kanamycin, tobramycin and gentamicin [17]. Currently, seven 16S rRNA methylase genes have been identified (*armA*, *rmtA*, *rmtB*, *rmtC*, *rmtD*, *rmtE*, *rmtF and npmA*), among which, *armA* and *rmtB* are the most common 16S rRNA methyltransferase genes [9,14,18,19].

Characterization and distribution of antimicrobial resistance gene profiles provide important information on the potential difficulty of treatment of bacteria. This information can be used to facilitate prompt and effective treatment of bacterial infections. In order to investigate the prevalence of aminoglycoside-resistance genes, several methods have been developed, including conventional single PCR and multiplex PCR assays combined with agarose gel electrophoresis analysis, hybridization with DNA probes, and sequence analysis [20,21]. Some drawbacks with these existing methods are time-consuming, labor-intensive, and difficult to analyze multiple genes simultaneously. DNA chips provide a versatile platform for rapidly screening several thousand potential antimicrobial resistance genes in parallel [22,23]. However, it is expensive and time-consuming for detecting numerous clinical isolates in the epidemiological investigation. So it is necessary to develop a rapid, cost effective and high throughput method to investigate the distribution of aminoglycoside resistance gene in clinical isolates.

The GenomeLab Gene eXpression Profiler genetic analysis system (GeXP analyzer) provided by Beckman Coulter Company (Brea, CA, USA) has been adopted by our group and successfully applied in the rapid detection of pandemic influenza A H1N1 virus [24], simultaneous detection of 11 human papillomavirus (HPV) genotypes [25], sixteen human respiratory virus types/subtypes [26] and nine serotypes of enteroviruses associated with hand, foot, and mouth disease [27] with high sensitivity and specificity. The general analysis procedure of GeXP assay consists of chimeric primer-based multiplex PCR amplification and capillary electrophoresis separation. In this study, a high throughput, cost-effective GeXP analyzer-based multiplex PCR assay (GeXP assay) was developed to simultaneously detect seven aminoglycoside- resistance genes, including five aminoglycoside-modifying enzymes genes [*aac(3)-II*, *aac(6′)-Ib*, *aac(6′)-II*, *ant(3″)-I* and *aph(3′)-VI*] and two 16S rRNA methyltransferase genes [*armA* and *rmtB*], and the results were compared with that of the conventional single PCR assay.

## Methods

### Bacterial strains

In order to evaluate the efficacy of primers and PCR assay, 8 reference strains listed in Table 1 (JN108884.1, JN119854.1, JN108899.1, HQ880271.1, GU944731.1, GU120473.1, JQ780837.1 and HQ880255.1) and 5 clinical strains (Table 2, including 3 strains of *Klebsiella pneumoniae* and 2 strains of *Escherichia coli*) were selected as positive controls, *Escherichia coli* ATCC#25922 and *Escherichia coli* J53 were used as negative controls. In the initial experiment, the distributions of aminoglycoside resistance genes among those controls strains were confirmed by conventional PCR with the specific primers listed in Table 3. Fifty six clinical isolates of Enterobacteriaceae were used to evaluate the utility of GeXP assay. All the clinical samples were taken as part of standard patient care from the inpatients at Guangzhou First Municipal People’s Hospital from January 2008 to December 2009. This protocol was approved by the Committee on the Use of Human Subjects in Research at Guangzhou First Municipal People’s Hospital, an affiliated hospital of Guangzhou Medical College. All the informed consents from the inpatients themselves or their guardians were obtained. In initial experiments, the identification of the clinical isolates and the minimum inhibitory concentrations (MICs) of antibiotics were confirmed by the VITEK® 2 system (bioMérieux, France) (Additional file 1). Forty eight of the 56 isolates (including 30 strains of *Klebsiella pneumoniae* and 18 strains of *Escherichia coli*) presented resistance to gentamicin (MIC≥16μg/mL), tobramycin (MIC≥16μg/mL) and/or amikacin (MIC≥64μg/mL), the other 8 isolates (including 5 strains of *Klebsiella pneumoniae* and 3 strains of *Escherichia coli*) were susceptible to gentamicin (MIC≤4μg/mL), tobramycin (MIC≤4μg/mL) and amikacin (MIC≤16μg/mL) according to the standards of Clinical and Laboratory Standards Institute (CLSI 2012).

**Table 1:** Distribution of aminoglycoside resistance genes in 8 reference strains

**Strains No.**	**Reference strains**	**Presence of aminoglycoside resistance genes**	**GenBank accession no.**
NF512663	*Escherichia coli*	*aac(6’)-Ib* [*aacA4*]*	JN108884.1
NF802568	*Escherichia coli*	*ant(3”)-II* [*aadA2*] *	JN119854.1
NF811738	*Klebsiella pneumoniae*	*aac(6’)-Ib* [*aacA4*] *&*ant(3”)-I* [*aadA1*] *	JN108899.1
NF707346	*Klebsiella pneumoniae*	*ant(2”)-I* [*aadB*] *&*ant(3”)-I* [*aadA1*] *	HQ880271.1
NF802824	*Klebsiella pneumoniae*	*acc(6’)-II*	GU944731.1
NF811834	*Klebsiella pneumoniae*	*aadA5*	GU120473.1
NF141160	*Acinetobacter baumannii*	*aac(3’)-I* [*aacC1*] * &*ant(3”)-I* [*aadA1*] *	JQ780837.1
NF910192	*Pseudomonas putida*	*aac(6’)-II & ant(2”)-I* [*aadB*] *	HQ880255.1

* Synonyms in the bracket.

**Table 2:** Distribution of aminoglycoside resistance genes in 5 positive control isolates

**Strains No.**	**Species**	**Presence of aminoglycoside resistance genes**						
		***aac(3)-II***	***aac(6’)-Ib***	***aac(6’)-II***	***ant(3”)-I***	***aph(3’)-VI***	***armA***	***rmtB***
1086	*Klebsiella pneumoniae*	+	+	-	+	+	+	+
1135	*Klebsiella pneumoniae*	+	-	+	+	+	+	+
1136	*Klebsiella pneumoniae*	+	+	+	+	+	+	-
1174	*Escherichia coli*	+	+	-	+	-	+	-
1313	*Escherichia coli*	+	+	-	+	+	+	-

### Bacterial genomic DNAs extraction and mono-resistance gene clone

The bacterial genomic DNA was extracted from overnight cultures using MiniBEST Bacterial Genomic DNA Extraction Kit Ver.2.0 (TAKARA, Dalian, China). The entire coding regions of *aac(3)-II*, *aac(6′)-Ib*, *aac(6′)-II*, *ant(3″)-I*, *aph(3′)-VI*, *armA* and *rmtB* were amplified individually from the positive control isolates with the specific primer listed in Table 3. PCR conditions for the amplifications were as follows: 5 min at 94°C; 30 cycles of 30 s at 94°C, 30 s at 56°C and 1 min at 72°C and a final extension of 5 min at 72°C. PCR products were cloned using the pMD18-T vector (TAKARA, Dalian, China), into E. coli JM109 and positive clones were selected using an X-Gal/IPTG LB agar plate containing ampicillin (100 mg/L). Recombinant plasmids were purified with QIAGEN Plasmid Mini Kit (Qiagen, Hilden, Germany), treated with the RNAse to eliminate residual RNA and subjected to DNA sequencing using T7 and SP6 sequence primers on an AB SOLiDTM 4.0 System (Applied Biosystems, USA). The obtained DNA sequences were compared with relevant sequences in the GenBank database by using the BLAST algorithm (http://blast.ncbi.nlm.nih.gov/Blast.cgi?PROGRAM=blastn&BLAST_PROGRAMS=megaBlast&PAGE_TYPE=BlastSearch&SHOW_DEFAULTS=on&LINK_LOC=blasthome).

**Table 3:** Primers for the entire coding regions of 7 aminoglycoside-resistance genes

**Primer**	**Sequence (5'→3')**	**Reference or source**	**Size (bp)**
*aac(3)-II*	F: ATATCGCGATGCATACGCGG	[31]	877
	R: GACGGCCTCTAACCGGAAGG		
*aac(6’)-Ib*	F: TTGCGATGCTCTATGAGTGGCTA	[32]	472
	R: CTCGAATGCCTGGCGTGTTT		
*aac(6’)-II*	F: CGACCATTTCATGTCC	This study*	542
	R: GAAGGCTTGTCGTGTTT		
*ant(3″)-I*	F: CATCATGAGGGAAGCGGTG	[33]	787
	R: GACTACCTTGGTGATCTCG		
*aph(3’)-VI*	F: ATGGAATTGCCCAATATTATT	[34]	780
	R: TCAATTCAATTCATCAAGTTT		
*armA*	F: CCGAAATGACAGTTCCTATC	[13]	846
	R: GAAAATGAGTGCCTTGGAGG		
*rmtB*	F: ATGAACATCAACGATGCCCTC	[13]	769
	R: CCTTCTGATTGGCTTATCCA		

*The primers have been validated with referenced strains (GU944731.1 and HQ880255.1).

### Primers

In this study, a total of one pair of universal primers (Tag-F/Tag-R) and seven pairs of chimeric primers (specific primers linked to the 3’ end to the universal primers) were designed (Table 4). Tag-F (AGGTGACACTATAGAATA) and Tag-R (GTACGACTCACTATAGGGA) were quasi-T7 sequences and selected by default using the GeXP eXpress Profiler software. The gene-specific sequences of the primers for *aac(3)-II, aac(6′)-II, ant(3″)-I* were previously reported [15,20]. The gene-specific sequences of other four pairs of primers were designed by NCBI Primer-Blast and GeXP eXpress Profiler softwares. The primer for *aac(6′)-Ib* also covered the variant gene *aac(6′)-Ib-cr* which not only resulted in aminoglycosides resistance but also mediated quinolone resistance [28]. The 5’ ends of the forward and reverse universal primers were labeled with fluorescent dye Cy5 and purified with high pressure liquid chromatography. All chimeric primers were purified by polyacrylamide gel electrophoresis.

**Table 4:** Primers information of GeXP assay

**Primer**	Sequence (5'→3')*	**GenBank accession no.**	**Position**	**Size (bp)**
*aac(3)-II*	F:AGGTGACACTATAGAATAACTGTGATGGGATACGCGTC	DQ449578.1	87359--87378	274
	R:GTACGACTCACTATAGGGACTCCGTCAGCGTTTCAGC**Y**A		87595--87576	
*aac(6’)-Ib*	F:AGGTGACACTATAGAATACTGTTCAATGATCCCGAGGT	JN861072.1	101468--101487	188
	R:GTACGACTCACTATAGGGATGGCGTGTTTGAACCATGTA		101619--101600	
*aac(6’)-II*	F:AGGTGACACTATAGAATATTCATGTCCGCGAGCACCCC	GU944731.1	1307--1326	215
	R:GTACGACTCACTATAGGGAGACTCTTCCGCCATCGCTCT		1485--1466	
*ant(3″)-I*	F:AGGTGACACTATAGAATATGATTTGCTGGTTACGGTGAC	HM106456.1	2207--2229	321
	R:GTACGACTCACTATAGGGACGCTATGTTCTCTTGCTTTTG		2490--2470	
*aph(3’)-VI*	F:AGGTGACACTATAGAATACGGAAACAGCGTTTTAGAGC	JF949760.1	727--746	288
	R:GTACGACTCACTATAGGGAGGTTTTGCATTGATCGCTTT		975--956	
*armA*	F:AGGTGACACTATAGAATATGCATCAAATATGGGGGTCT	FJ410928.1	3953--3972	247
	R:GTACGACTCACTATAGGGATGAAGCCACAACCAAAATCT		4162--4143	
*rmtB*	F:AGGTGACACTATAGAATAGCTGTGATATCCACCAGGGA	FJ410927.1	5326--5345	177
	R:GTACGACTCACTATAGGGAAAGCTTAAAAATCAGCGCCA		5465--5446	
Cy5-labled Tag	F:AGGTGACACTATAGAATA			
	R:GTACGACTCACTATAGGGA			

*Universal tag sequences are underlined.

### Evaluation of the specificity of the GeXP assay

**Figure 1:**
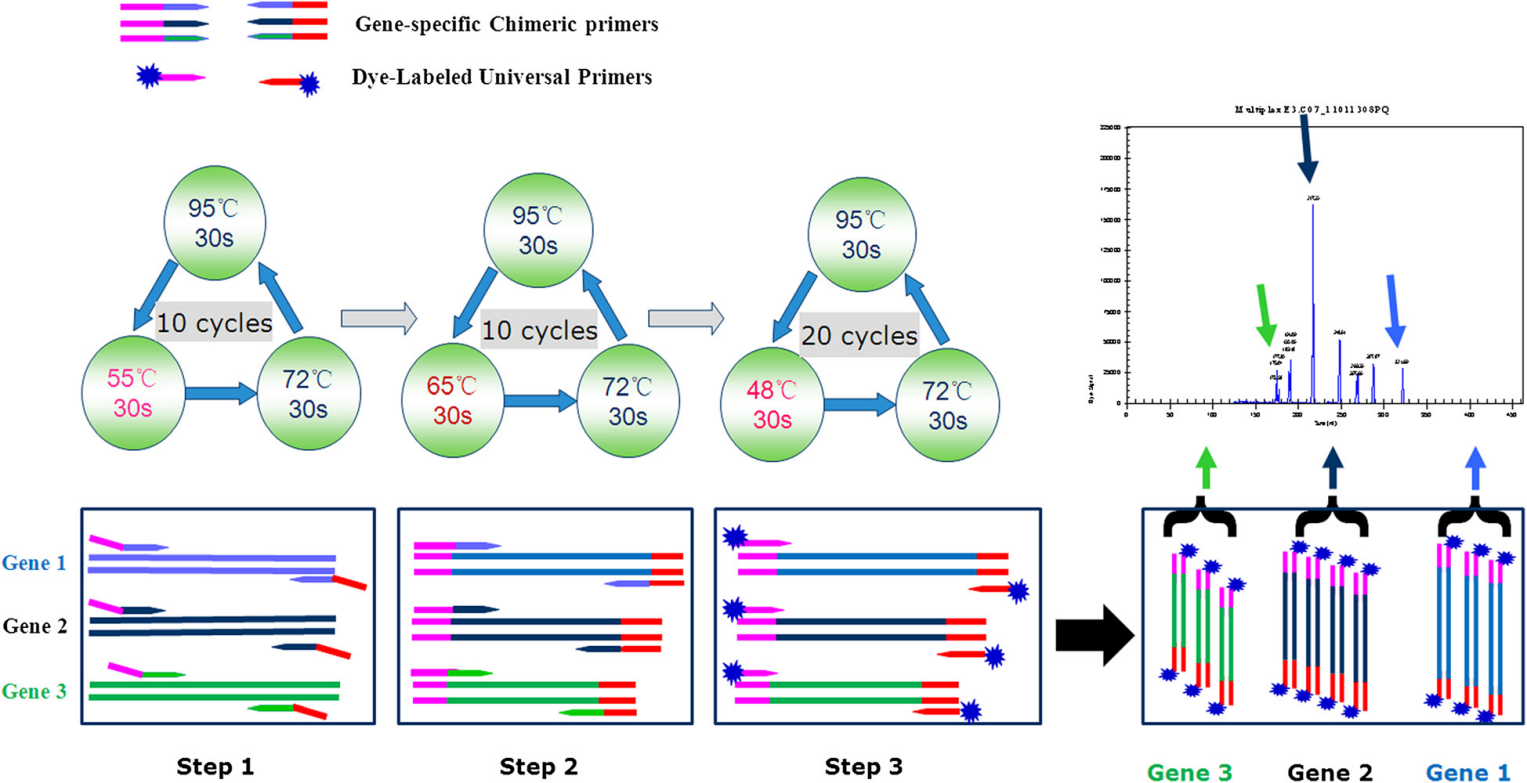
**Diagram of the analysis procedure of GeXP assay.** The analysis procedure of GeXP assay consists of chimeric primer-based multiplex PCR amplification and capillary electrophoresis separation. The principle of GeXP multiplex amplification assay is based on the amplification of two sets of primers: the gene-specific chimeric primers and the dye-labeled universal primers. After amplified fragments were separated, the peaks of genes were analyzed and reported on the electropherogram, respectively.

The DNA templates were extracted bacterial genomic DNAs of the 8 reference strains, 5 positive control isolates, 2 negative controls and 7 recombinant plasmids harboring each of the 7 resistance genes, respectively. The mono GeXP assay and GeXP assay were developed using single template and each pair of gene-specific primers (for mono GeXP assay) or using single template in a multiplex primer format (for GeXP assay), respectively, to ascertain the actual amplicon size of each target region. The PCR assays were performed with QIAGEN Multiplex PCR kit (Qiagen, Hilden, Germany) in a 25 μl volume containing 12.5 μl of 2× QIAGEN Multiplex PCR Master Mix (HotStarTaq® DNA Polymerase, Multiplex PCR Buffer, dNTP Mix) and 1 μl of DNA templates. The mono GeXP assay contained 50 nM of each pair of gene-specific chimeric primers individually while the GeXP assay contained 50 nM of each of 7 pairs of gene-specific chimeric primers and 500 nM of the universal Tag primers as the final concentrations, nuclease-free water was added to 25 μl reaction volume. The PCR was performed under the following conditions: 95°C for 10 min, followed by three steps of amplification procedures reaction according to the temperature switch PCR (TSP) strategy [29]: step 1, 10 cycles of 95°C for 30 s, 55°C for 30 s, and 72°C for 30 s; step 2, 10 cycles of 95°C for 30 s, 65°C for 30 s, and 72°C for 30 s; step 3, 20 cycles of 95°C for 30 s, 48°C for 30 s, and 72°C for 30 s (Figure 1).

### Separation by capillary electrophoresis (CE) and fragment analysis

PCR products were combined with DNA Size Standard at the volume ratio of 2: 0.25 per reaction in 25 μl of Sample Loading Solution and separated on a GeXP Analyzer by capillary electrophoresis, following the protocols as described previously [27,30]. After amplified fragments were separated, the peaks were initially analyzed using the Fragment Analysis module of the GeXP system software and matched to the appropriate amplified products. The peaks height for each gene was reported in the electropherogram, respectively (Figure 1). The dye signal strength was measured by fluorescence spectrophotometry in arbitrary units (A.U.) of optical fluorescence. For all amplified products, the reaction was considered positive when the value of dye signal was over 1000 A.U. In addition, PCR products were sequenced and compared with relevant sequences in the GenBank database by using the BLAST algorithm (http://blast.ncbi.nlm.nih.gov/Blast.cgi?PROGRAM=blastn&BLAST_PROGRAMS=megaBlast&PAGE_TYPE=BlastSearch&SHOW_DEFAULTS=on&LINK_LOC=blasthome).

### Evaluation of the limit of detection of the GeXP assay

The limit of detection of GeXP assay was measured by using 7 purified recombinant plasmids containing seven complete resistance genes, respectively. The concentration for each resistance gene was quantitated by spectrophotometry (NanoDrop ND-2000) and serial ten-fold diluted from 10^4^ copies to 1 copy per microliter, and then individually subjected to the GeXP assay. The concentrations of specific primers were then optimized according to the amplification efficiency of the GeXP assay using single template. The sensitivity of the optimized GeXP assay for simultaneous detection of seven genes was re-evaluated using pre-mixed recombinant plasmids containing seven resistance genes ranging from 10^4^ copies to 1 copies for each resistance gene per microliter for three times on three different days.

### Application to clinical isolates

Genomic DNAs extracted from 56 clinical isolates were used to illustrate the clinical performance of the optimized GeXP assay. All the clinical isolates were detected in parallel by conventional single PCR with the specific primers reported by the previous study [13,31-35]. The amplified products were analyzed by electrophoresis at 100 V for 25 to 30 minutes in a 2% agarose gel stained with SYBR green. Positive PCR products were purified, sequenced using T7 and SP6 sequence primers on AB SOLiDTM 4.0 System (Applied Biosystems, USA) and compared with the sequences in GenBank for gene type identification by using the BLAST algorithm.

### Statistical analysis

All statistical analyses were performed using Statistical Package for Social Sciences (SPSS) software (version 13.0) for Windows. The *x*^2^-test and Fisher’s exact test were conducted to measure the detection agreement of GeXP assay with conventional single PCR method.

## Results and discussion

In this study, we adopted seven pairs of chimeric gene-specific primers to develop a GeXP assay for simultaneous detection of seven common aminoglycoside-resistance genes including five aminoglycoside-modifying enzymes genes [*aac(3)-II*, *aac(6′)-Ib*, *aac(6′)-II*, *ant(3″)-I* and *aph(3′)-VI*] and two 16S rRNA methyltransferase genes [*armA* and *rmtB*].

The principle of proposed GeXP assay is based on the amplification with two sets of primers: the universal primers and the gene-specific chimeric primers (gene-specific primers linked to the 3’ ends of universal primer sequences). During the first few cycles of PCR, amplification is carried out by chimeric forward and reverse primers. In later stages of PCR, amplification is predominantly carried out by universal forward and reverse primers. All gene targets in the multiplex panel are amplified by the correspondent chimeric primers and the universal primers. The universal primer is fluorescently dye-labeled enabling subsequent fluorescence detection of amplicons by capillary electrophoresis. The temperature switch PCR (TSP) strategy was adopted to optimize the amplification parameters. The triphasic PCR parameters of the TSP allow a multiplex PCR to be performed under standardized PCR conditions, and therefore do not require optimization of each individual PCR assay. The optimal settings for three different denaturation temperatures and the amplification cycle conditions were determined in the current protocol. The concentration of the fluorescently dye-labeled universal primers was almost ten times that of the chimeric primers in the GeXP assay, so in the last 20 cycles of PCR, amplification was carried out predominantly with universal forward and reverse tag primers (Figure 1). This should reduce the occurrence of preferential amplification in the reaction and minimize nonspecific reactions.

### Evaluation of the specificity of the GeXP assay

**Figure 2:**
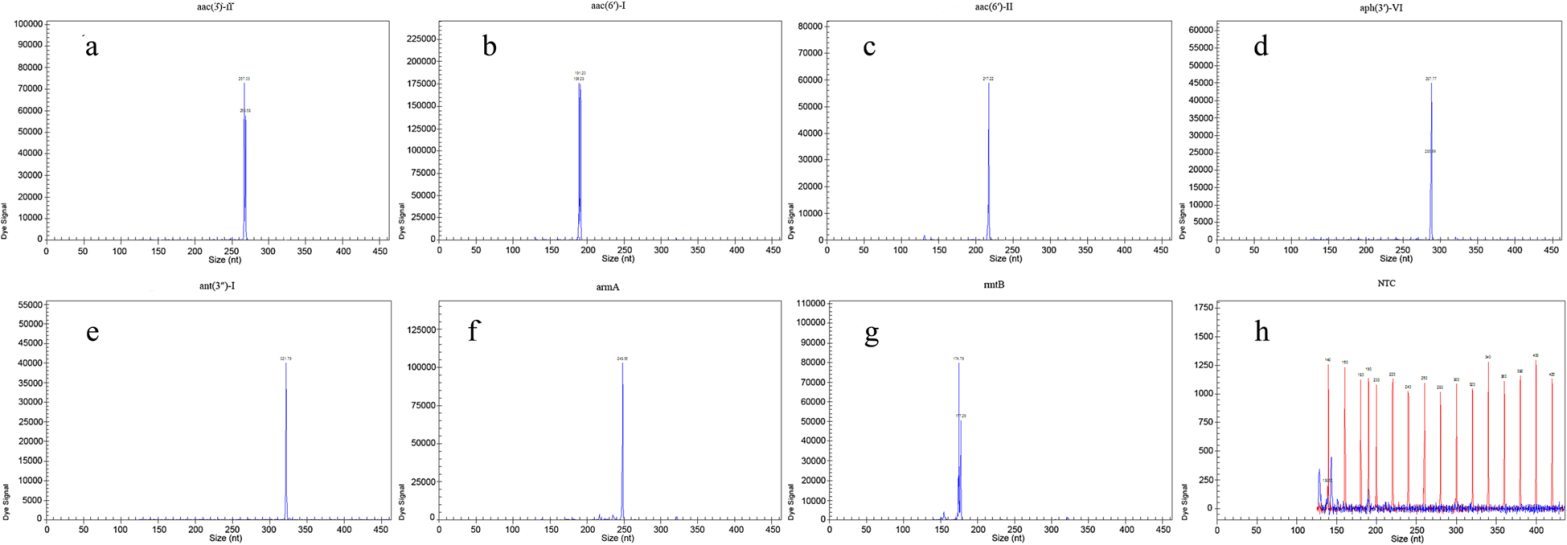
**The specificity of GeXP assay for the detection of aac(3)-II, aac(6′)-Ib, aac(6′)-II, ant(3″)-I, aph(3′)-VI, armA and rmtB.** Seven recombinant plasmids harboring aminoglycoside-resistance genes were respectively detected via the GeXP assay. All the specific peaks were observed presenting the gene-specific target amplicons of *aac(3)-II, aac(6′)-Ib, aac(6′)-II, ant(3″)-I, aph(3′)-VI, armA* and *rmtB*, respectively (**a**~**g**). The negative control assay clearly showed the DNA size standard from 140 to 420 nt (peaks in red color) without nonspecific products presented (**h**).

In mono GeXP assay, each pair of gene-specific primers could amplify the target region of the corresponding aminolycoside resistance gene without nonspecific products. The amplicon size for each target resistance gene was as follows, *aac(3)-II*: 267-269 bp, *aac(6′)-Ib*: 189-191 bp, *aac(6′)-II*: 217-218 bp, *ant(3″)-I*: 320-322 bp, *aph(3′)-VI*: 286-288 bp, *armA*: 248-249 bp and *rmtB*: 174-177 bp. In GeXP assay using seven recombinant plasmids as templates, all the specific amplification peaks were observed presenting the gene-specific target amplicon without cross-amplification (Figure 2). In GeXP assay using 8 reference strains and 5 positive control strains as templates, all the correspondent genes in this study could be detected without nonspecific amplification. The other aminoglycoside resistance genes (e.g., *ant(2”)-I* and *aadA5*) which were not targeted in this study did not generate nonspecific amplification in the GeXP assay. All the amplicons of mono GeXP assay were sequenced and confirmed as the target genes by BLAST comparison with relevant sequences in the GenBank database (data not shown).

### Evaluation of the analytic sensitivity of the GeXP assay

**Figure 3:**
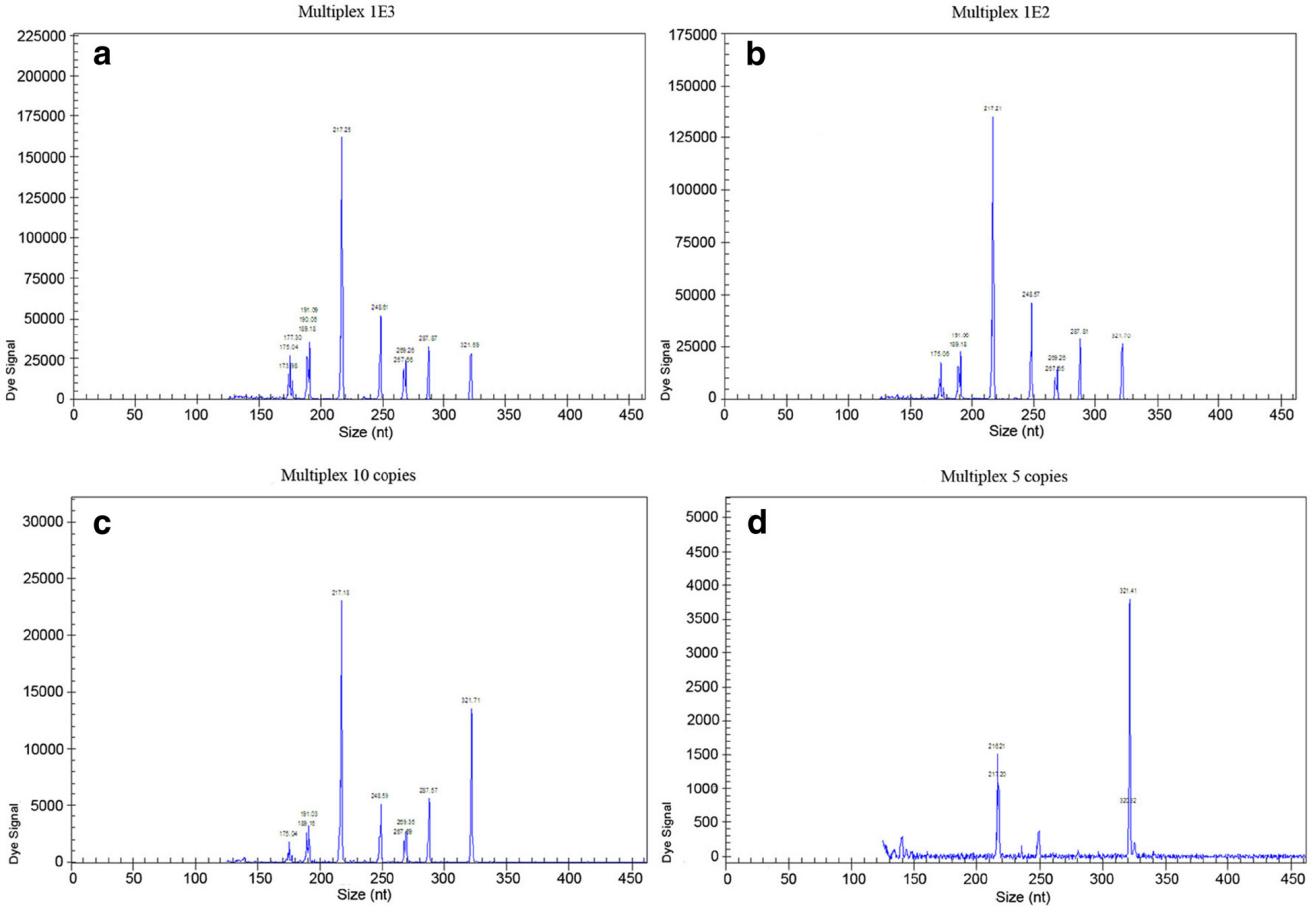
**The sensitivity of GeXP assay for detection of seven aminoglycoside-resistance genes.** The GeXP assay was carried out using different concentrations of seven premixed recombinant plasmids with 1000 copies (**a**), 100 copies (**b**), 10 copies (**c**) and 5 copies (**d**), respectively. All of seven aminoglycoside-resistance genes could be detected at 1000, 100 and 10 copies levels (**a**, **b** and **c**); only *aac(6′)-II* (217 bp) and *ant(3″)-I* (321 bp) could be detected at 5 copies levels in the optimized GeXP assay (**d**).

The sensitivity of the GeXP assay was measured using quantitative recombinant plasmids. The GeXP assay with 50 nM of each pair of gene-specific chimeric primers could individually detect as few as 5 copies of *armA*, 10 copies of *aac(3)-I*, *aac(6′)-Ib* and *rmtB*, about 100 copies of *aac(6′)-II, aph(3′)-VI* and *ant(3″)-I* per reaction. Based on all the amplification efficiency (above analytic sensitivity results) of GeXP assay with single recombinant plasmid template, the concentration of each chimeric primer in the optimized GeXP assay was adjusted as follows: the primers concentrations of *aac(3)-II*, *aac(6′)-Ib*, *armA* and *rmtB* were 50 nM, while the concentrations of the other three pairs of chimeric primers [including *aac(6′)-II*, *aph(3′)-VI* and *ant(3″)-I*] were doubled up to 100 nM. The optimized GeXP assay reduced the potential interference due to the preferred amplification in mixed settings and achieved a sensitivity of 10 copies when seven pre-mixed recombinant plasmids templates were present in three independent experiments on three different days (Figure 3).

### Application to clinical specimens and statistical analysis

Fifty six strains of Enterobacteriaceae were detected simultaneously by both the GeXP assay and the conventional single PCR followed by electrophoresis analysis in a 2% agarose gel. The distribution of aminoglycoside resistance genes detected by GeXP assay in 56 clinical isolates was shown in Additional file 1. All the sequenced amplicons of both assays were confirmed as true target genes by comparing with relevant sequences in the GenBank database (data not shown). All of the 48 resistant isolates (30 strains of *Klebsiella pneumoniae* and 18 strains of *Escherichia coli*) harbored at least one modifying enzyme gene and one or two 16S rRNA methylase genes. None of the 8 susceptible isolates harbored these resistance genes by both assays. In comparison with the results of conventional single PCR, the sensitivities of the GeXP assay for detection of *aac(3)-II*, *aac(6′)-Ib*, *aac(6′)-II*, *ant(3″)-I*, *aph(3′)-VI*, *armA* and *rmtB* were 92.50% (37/40), 100% (11/11), 88.89% (8/9), 100% (40/40), 83.33% (10/12), 95.24% (40/42) and 93.33% (14/15),respectively, and the specificities were 88.23% (15/17), 93.33% (42/45), 95.74% (45/47), 87.50% (14/16), 100% (44/44), 85.71% (12/14) and 92.68% (38/41), respectively. The Kappa values of the GeXP assay and conventional single PCR for detecting the seven resistance genes were 0.831, 0.846, 0.810, 0.909, 0.887, 0.810 and 0.825, respectively. Those specimens that were negative by the conventional single PCR but positive by the GeXP assay (Table 5) were confirmed later by mono-GeXP assay and sequenced to be true positives, suggesting the optimized GeXP assay performed a better sensitivity than the conventional method. Meanwhile, some genes were detected as positive by conventional method but negative by multiplex GeXP assay (4^th^ column of Table 5). The false negative genes of multiplex GeXP assay could be detected by mono-GeXP assay with less than 2000 A. U. dye signal strength of the peaks in these false negatives. The later analysis of the distribution of seven aminoglycoside-resistance genes showed that all of false negatives of multiplex GeXP assay harbored more than four genes, and the concentration of each gene in these isolates largely varied, suggesting the false negatives of multiplex GeXP assay were missed due to the ignorance of the lower peak (less than 2000 A. U. dye signal strength) overcast by higher peaks (more than 100000 A. U.).

**Table 5:** Comparison of GeXP assay and conventional single PCR for detecting seven aminoglycoside-resistance genes

**Resistance genes**	**GeXP assay**	**Conventional single PCR**			**Measures of agreement**
		**Positive**	**Negative**	**Total**	Kappa values*
*aac(3)-II*	Positive	37	2	39	0.831 (*P*=0.000)
	Negative	2	15	17	
	Total	39	17	56	
*aac(6')-Ib*	Positive	11	3	14	0.846 (*P*=0.000)
	Negative	0	42	42	
	Total	11	45	56	
*aac(6')-II*	Positive	8	2	10	0.810 (*P*=0.000)
	Negative	1	45	46	
	Total	9	47	56	
*ant(3″)-I*	Positive	40	2	42	0.909 (*P*=0.000)
	Negative	0	14	14	
	Total	40	16	56	
*aph(3')-VI*	Positive	10	0	10	0.887 (*P*=0.000)
	Negative	2	44	46	
	Total	12	44	56	
*armA*	Positive	40	2	42	0.810 (*P*=0.000)
	Negative	2	12	14	
	Total	42	14	56	
*rmtB*	Positive	14	3	17	0.825 (*P*=0.000)
	Negative	1	38	39	
	Total	15	41	56	

*As a rule of thumb, values of Kappa from 0.40 to 0.59 are considered moderate, 0.60 to 0.79 substantial, and 0.80 outstanding.

The GeXP assay in our study can simultaneously detect 7 genes related to resistance to aminoglycosides. The cost is about 5£ per test, versus 5£ using commercial real-time PCR kit for individual gene in a single PCR assay. The whole reaction was completed in one tube within 2 hours, followed by capillary electrophoresis separation on the GeXP analyzer in 45 min, so the total turn around time (TAT) is less than 3 hours, versus 12-24 hours by antibiotic susceptibility test (AST). This strategy of multiplex PCR amplification would be used to detect more resistance genes with high sensitivity and specificity. In addition, two 96-well plates can be placed in parallel in a GeXP machine at the same time, which can be combined with the automation workstation to further increase the throughput of the samples.

## Conclusions

The GeXP assay is a time-saving, cost-effective and high throughput method with high sensitivity and specificity for simultaneously detecting seven common aminoglycoside-resistance genes. Further improvement by large-scale studies for determination of the sensitivity, specificity, and clinical utility of this new method will be needed before GeXP assay can be implemented effectively in routine testing environments for molecular epidemiologic survey of resistance genes and offer a directory suggestion for clinical antibiotic therapy.

## Competing interests

The authors declare that they have no competing interests.

## Authors’ contributions

We warrant that all authors have seen and approved the manuscript and they have contributed significantly to the work. XH, BX, and YY were involved in the operation of GeXP experiment and collection of the clinical specimens, DL, MY, JW and HS offered great help in the evaluation of GeXP results using conventional methods. XZ and XM designed and coordinated the study, analyzed data. XH, XZ and XM drafted the manuscript. All authors read and approved the final manuscript.

## Supplementary Material

Additional file 1 Minimal inhibitory concentration of antimicrobials and distribution of aminoglycoside resistance genes in 56 clinical isolates. Click here for file
